# Adjustments disorders among residents, burnout and depression

**DOI:** 10.1192/j.eurpsy.2025.1985

**Published:** 2025-08-26

**Authors:** S. V. Kuzmina, A. D. Shurigina

**Affiliations:** 1 psychiatry and medical psychology, Kazan state medical university; 2 Republican Psychiatry hospital, Kazan, Russian Federation

## Abstract

**Introduction:**

In 2022, studies were conducted, the results of which revealed that nursing staff are at greater risk of developing anxiety disorders, and the development of these symptoms is influenced by work schedule and workload level. Also, do not forget about the relationship between anxiety and affective disorders and emotional burnout syndrome (EBS), which is important in diagnosis

**Objectives:**

The purpose of the study is to evaluate the role of the phenomenon of burnout as a predictor of the development of anxiety and depression based on a comprehensive assessment of EBS and anxiety-affective symptoms among medical residents; identify risk factors for the development of EBS.

**Methods:**

A questionnaire including socio-demographic characteristics (gender, age, place of residence), the Maslach Burnout Inventory (MBI) questionnaire; questionnaire scale: PHQ-9 (via Google platform); clinical interviewing was carried out among 98 residents

**Results:**

two profiles of respondents – surgical (57.1%) and therapeutic (42.9%), 73.5% - female, 26.5% - male. The majority were in the age range from 25-29 years (77.6%). The most closely related to burnout is the time spent working at the clinical base and the workload outside of residency. 21% have mild depression, 24% have moderate depression, and 10% have severe depression, which further requires the identification of a correlation between two indicators of EBS and affective symptoms; 31.6% have severe emotional burnout, which affects education and the quality of medical care provided.

**Conclusions:**

The question of the relationship between EBS and depressive symptoms remains open. In our simultaneous study, the relationship between the burnout index and the results of PHQ-9 is traced, which may suggest that EBS is considered as a risk factor for the development of affective symptoms of depression and requires further investigation of the problem It should be noted the importance of introducing digital technologies for the examination of employees and the timely detection of signs of EBS

**Disclosure of Interest:**

None Declared

